# De-Escalation Strategies for Human Papillomavirus-Associated Oropharyngeal Squamous Cell Carcinoma—Where Are We Now?

**DOI:** 10.3390/curroncol29050295

**Published:** 2022-05-18

**Authors:** Jennifer A. Silver, Sena Turkdogan, Catherine F. Roy, Thavakumar Subramaniam, Melissa Henry, Nader Sadeghi

**Affiliations:** 1 Department of Otolaryngology—Head and Neck Surgery, McGill University Health Centre, Montreal, QC H4A 3JI, Canada; jennifer.silver2@mail.mcgill.ca (J.A.S.); sena.turkdogan@mail.mcgill.ca (S.T.); catherine.roy6@mail.mcgill.ca (C.F.R.); thavakumar.subramaniam@mail.mcgill.ca (T.S.); 2 Department of Otolaryngology—Head and Neck Surgery, McGill University, Montreal, QC H4A 3JI, Canada; melissa.henry@mcgill.ca; 3 Gerald Bronfman Department of Oncology, McGill University, Montreal, QC H4A 3JI, Canada; 4 Lady-Davis Institute for Medical Research, Montreal, QC H3T 1E2, Canada; 5 Segal Cancer Centre, Jewish General Hospital, Montreal, QC H3T 1E2, Canada; 6 Research Institute of McGill University Health Center, McGill University, Montreal, QC H4A 3JI, Canada

**Keywords:** oropharyngeal squamous cell carcinoma, human papillomavirus, de-escalation, transoral surgery

## Abstract

The prevalence of oropharyngeal squamous cell carcinoma has been increasing in North America due to human papillomavirus-associated disease. It is molecularly distinct and differs from other head and neck cancers due to the young population and high survival rate. The treatment regimens currently in place cause significant long-term toxicities. Studies have transitioned from mortality-based outcomes to patient-reported outcomes assessing quality of life. There are many completed and ongoing trials investigating alternative therapy regimens or de-escalation strategies to minimize the negative secondary effects while maintaining overall survival and disease-free survival. The goal of this review is to discuss the most recent advancements within the field while summarizing and reviewing the available evidence.

## 1. Introduction

Worldwide, there were 900,000 new diagnoses of head and neck cancers and 400,000 deaths from their cancers in 2020 [1]. In the United States, the overall incidence of head and neck cancers has been decreasing due to lower rates of tobacco consumption, however, not all subtypes are following this trend [2,3]. The prevalence of oropharyngeal squamous cell carcinoma (OPSCC) has been increasing in North America due to human papillomavirus (HPV)-associated disease [2]. Recent studies have found that approximately 60–70% of OPSCC cases in the United States are associated with HPV, in contrast to traditional tobacco- and alcohol-related OPSCC [2,4,5,6,7,8,9].

Patients with HPV-associated OPSCC are molecularly and clinically distinct as compared with those with conventional OPSCC. The etiology arises from the double-stranded DNA viruses E6 and E7 oncogenes that inactivate the p53 tumor suppressor gene and the retinoblastoma protein which lead to release of transcription factors causing cell cycle progression [10]. Patients with HPV-associated OPSCC are younger, healthier at baseline, often with minimal or no tobacco exposure, and have a more favorable prognosis with standard treatment, demonstrated in retrospective and prospective research [6,8,11]. The improved survival rate of HPV-associated OPSCC has resulted in important changes to the American Joint Committee on Cancer (AJCC) staging system. In 2017, the eighth edition categorized oropharyngeal cancers by p16 status, a surrogate marker for HPV status, downstaging the p16 positive OPSCC from the prior edition [12].

Historically, OPSCC treatments have primarily consisted of radiation-based therapies, which were favored over the invasive and often disfiguring open surgical approaches with high morbidity and mortality [13]. It has been shown that treatment intensification with concurrent chemoradiation (CRT) improves overall survival of head and neck cancer patients as compared with radiotherapy alone [14,15,16,17].The first meta-analysis on this subject was performed in 2000 and was updated in 2009. The versions both concluded that concomitant CRT provided a five-year overall survival benefit as compared with locoregional treatment with radiotherapy alone at 4% and 4.5%, respectively. In analyses based on tumor subsite, they concluded that there was a benefit for concurrent CRT for OPSCC. Blanchard et al. conducted another meta-analysis and concluded that OPSCC patients had a five-year survival benefit of 5.3% and that concomitant chemotherapy to locoregional treatment was the most efficacious timing of administration [14].

The standard treatment regimens for OPSCC patients currently consists of surgery with a preference for minimally invasive transoral surgery, radiation therapy (RT), and chemotherapy as either single modality or multimodality based on TNM staging. For stages I and II OPSCC, treatment often consists of surgery or RT, while stages III and IV OPSCC are often treated with concomitant CRT or surgery with adjuvant RT or CRT based on pathological features [18].

With a five-year survival rate greater than 80%, the younger and healthier patients in remission from HPV-associated OPSCC are living longer with post-treatment toxicities [8,19]. The three treatment modalities, i.e., radiation therapy, chemotherapy, and surgery, have specific benefits and both short-term and long-term side effects to consider.

### 1.1. Radiation

As described above, early stage OPSCC can be treated with single modality RT. More advanced cancers are treated with multimodality treatment which includes RT. Early complications include dermatitis of varying degrees, pain, mucositis, dysphagia, and infection. However, it is the late post-RT consequences that are often debilitating and affect quality of life in the decades after treatment. These are well described in the literature, and include xerostomia due to damage to the salivary glands, trismus from contraction and fibrosis of the masticator muscles, and less frequently osteoradionecrosis which may lead to infection, fracture and fistula formation, rare ischemic stroke, and second primary radiation-induced malignancy [20,21]. The radiation toxicities have been proven to be augmented in patients also receiving concurrent chemotherapy [8,22]. Efforts have been made to reduce RT-based adverse effects with newer technologies such as intensity-modulated radiotherapy (IMRT) or intensity-modulated proton therapy (IMPT), sparing swallowing and salivary gland structures, and lowering doses of RT with some success [23].

### 1.2. Chemotherapy

Chemotherapy can be administered as induction prior to definitive therapy, concomitantly with RT, or as an adjuvant systemic therapy. Neoadjuvant chemotherapy has potential benefits, including tailoring which definitive treatment to offer the patient if there is response to chemotherapy (radical surgery or concomitant CRT), facilitating organ preservation, providing systemic therapy for micro-metastases, and providing initial locoregional treatment while preparing for radiation [24,25,26].

Chemotherapy toxicity is mediated by anti-mitotic, cytotoxic, or photosensitizing properties, as well as their myelo- and immuno-suppressive effects. Haematologically, chemotherapy agents inducing neutropenia puts patients at risk of systemic infections caused by viral, bacterial, and fungal organisms, where chemotherapy-induced significant mucositis and stomatitis may be the site of entry [27]. Anemia and thrombocytopenia may additionally increase the risk of bleeding complications. In head and neck cancer of all sites, neoadjuvant chemotherapy followed by CRT has been shown to not have any added benefit for overall survival over concurrent CRT in the DeCIDE study [28]. However, a sub-analysis in this study showed a trend towards improved survival for patients with HPV-associated OPSCC. The neoadjuvant chemotherapy regimen used in the DeCIDE study included 5-florouracil (5-FU) along with docetaxel and cisplatin. Furthermore, the concomitant chemotherapy included docetaxel, hydroxyurea, and 5-FU, significantly adding to the intensity and toxicity of the treatment which was not warranted in HPV-associated OPSCC. A common regimen for HPV-associated OPSCC is docetaxel and cisplatin, which is generally well tolerated [29]. Relatively recently, 5-fluorouracil has been eliminated in the chemotherapy regimen as it brought significant toxicity. Without it, there has been better patient tolerance while maintaining the oncological effect [29]. The significance of good oncological efficacy of chemotherapy has been demonstrated in studies by Sadeghi et al. where neoadjuvant chemotherapy was administered in HPV-associated OPSCC patients and demonstrated pathologic complete response in 72% and 57%, respectively, for the primary tumor and the cervical nodal disease [29,30].

### 1.3. Surgery

The great benefit of surgery is histopathological assessment of the tumor to guide further treatments. Analysis of the specimen can identify perineural invasion, extracapsular spread, angioinvasion, microscopic disease, and positive margins. Depending on the risk stratification, observation, adjuvant RT or adjuvant CRT may be recommended to the patient [31].

Surgical resection can be further divided into open or transoral approaches and have different advantages, risks, and long-term morbidities associated with them. Open surgical approaches include trans-mandibular and trans-pharyngeal routes and often require microvascular free flap reconstruction based on the defect size. A complication rate of 50% or more has been reported in the literature in open approaches [32,33]. Potential complications include damage to nerves and vessels, disfigurement, scarring, dysphagia and aspiration, speech articulation difficulties, trismus, and malocclusion. A tracheostomy is required in the vast majority of patients for airway protection, and time to decannulation and time to adequate oral intake is lengthened often requiring permanent or transitory tracheostomy and percutaneous gastrostomy feeding at home.

Transoral surgical techniques include both transoral laser microsurgery (TLM) and transoral robotic surgery (TORS). The limiting factors for transoral surgery are difficult exposure causing poor visualization of the primary tumor to obtain adequate margins; surgeon-dependent training and skills for these techniques; and availability or access to the necessary equipment, lasers, or robotic surgical systems. However, when possible, it is used in T1 and T2 tumors, and resections in difficult-to-reach areas such as the base of tongue and vallecula are now attainable [34,35]. Benefits include less functional disability and dysphagia, no cosmetic deformity, lower tracheostomy and percutaneous gastrostomy rates, and higher rates of decannulation when tracheostomy is necessary [36,37].

## 2. De-Escalation Strategies

With the rise of HPV-associated OPSCC and its lower mortality rates, younger and healthier patients in remission are living longer with the morbidities afflicted by their treatments. In Ang et al.’s publication of the RTOG 0129 trial, the prognosis of OPSCC patients was sub-analyzed by HPV status [8]. Patients with HPV-associated disease had higher overall survival rates at three years (82.4% vs. 57.1%) and progression-free survival rates (73.7% vs. 43.4%). The HPV-associated cohort was less likely to smoke and had a lower cumulative pack-years of smoking exposure. This research identified cigarette use as an independent prognostic factor for both HPV-positive and -negative head and neck cancers, with a 1% increase in risk of death or relapse with each additional pack-year of smoking. A history of 10 pack-years has been identified as a cut-off point for impact on survival. Therefore, those with HPV-associated OPSCC without or with minimal tobacco history are considered to be low risk.

The oropharynx is an anatomical region essential for daily functions, such as speech and deglutition, as well as sensation and emotional expression. Different treatments can limit these activities of daily living and greatly affect quality of life. Maintaining or prioritizing functional quality of life can greatly improve a patient’s outlook on their cancer diagnosis. Given the improved prognosis of HPV-associated OPSCC, studies have shifted from mortality-based outcomes to patient-reported outcomes assessing quality of life [31]. Therefore, the emphasis has been placed on identifying alternative therapy regimens or de-escalation strategies to minimize negative secondary effects while maintaining overall survival and disease-free survival. This review will explore different de-intensification studies and clinical trials that are seeking to improve the quality of life of post-treatment HPV-associated OPSCC patients without compromising survival (Table 1).

## 3. Upfront Surgery and Pathology-Based Adjuvant Therapy Approach

Since the advent of TORS, primarily indicated and approved for T1 and T2 oropharynx cancer, there has been a significant shift in the management of early OPSCC towards TORS and neck dissection. This has been followed by risk-based adjuvant RT or CRT based on pathology. The shift in treatment has been based on successful oncologic and swallowing outcomes based on case series [38,39].

ORATOR (NCT01590355 (accessed on 1 January 2022)) is a Canadian-based trial randomizing patients with AJCC 7th edition T1–2, N0–2 OPSCC to either upfront surgical resection via TORS and neck dissection with risk-based adjuvant CRT (*n* = 34) or RT at 70 Gy with concurrent chemotherapy if node or margin positive (*n* = 34). High-dose cisplatin was used; however, patients received cetuximab or carboplatin regimens if not fit for high-dose cisplatin [40,41]. Eighty-eight percent of patients had HPV-associated OPSCC. In the surgical arm, 10 patients underwent primary surgery without adjuvant therapy, 16 patients received adjuvant RT (60 Gy), and 8 patients underwent adjuvant CRT (64 Gy RT and 5 cisplatin, 3 carboplatin). Among the 34 patients in the RT group, two withdrew from the study, 9 patients received RT alone, and 23 patients underwent concurrent CRT, with 4 patients requiring salvage surgery. The main endpoint studied was swallowing-related quality of life, as measured by the MD Anderson Dysphagia Inventory (MDADI) standardized questionnaire, with other quality of life scales used as well. Their initial results at a median follow-up of 27 months demonstrated a greater swallowing-associated quality of life in the RT group after one year, although this value was not clinically significant, as the threshold of a meaningful difference between groups must be a 10-point score discrepancy [40]. However, a recent update regarding long-term results at median follow-up time of 45 months published in January 2022 demonstrated that this difference between groups in swallowing quality of life decreases over time and the oncological outcomes are similar [41]. Overall, the main differences within the treatment arms are the distinct side-effect profiles. The RT arm patients have greater ototoxicity and neutropenia while the surgical group has greater pain, trismus, and bleeding. In the RT group, one patient required a percutaneous feeding tube at one year, but at two and three years had a total oral diet without restrictions. In the surgical arm, no patients needed a percutaneous feeding tube at one year, however, one patient who underwent post-operative CRT had a decline in swallowing capacity, and at 30 months post-treatment required insertion of a feeding tube. Recurrence rates were similar in each group, at four patients per treatment arm. The authors ultimately concluded that both are reasonable treatment options of T1–2, N0–2 OPSCC and should be discussed openly to allow the patient to weigh the risks and benefits of the different modalities [41]. ORATOR attempted to compare upfront surgery to radiation-based therapy, however, risk stratification and adjuvant treatment allocation lead to 47% of patients in this group requiring adjuvant RT and an additional 24% of patients to requiring CRT post-operatively. This surgery-first approach carries a high risk of post-operative adjuvant therapy, which leads to additional locoregional therapy instead of treatment de-escalation. In essence, patients in this trial were treated with a high rate of adjuvant therapy (71% of patients), which does not allow for a simplified comparison of surgery versus RT.

This same group conducted ORATOR2 (NCT03210103 (accessed on 1 January 2022)), where they compared de-intensified treatments in early stage disease (T1–2, N0–2, HPV-associated OPSCC, AJCC 8th edition) [42]. Patients were risk-stratified by smoking status and randomized to primary transoral surgery and neck dissection and lowered dose adjuvant RT if needed ± chemotherapy or de-escalated RT af 60 Gy ± chemotherapy [43]. These two treatment arms are based on other trials that will be discussed in this review (*E3311* and *NRG HN002*). The primary endpoint was overall survival and secondary endpoints were progression-free survival, quality of life, and toxicity. 61 patients were recruited (31 in TORS and neck dissection arm and 30 in RT arm) before this trial was closed early due to treatment-related deaths in two patients (bleeding and osteomyelitis after RT, both in the surgical arm). Median follow-up was 17 months. The two-year overall survival estimates in the TORS group versus RT group for were 89.1% and 100% respectively. The two-year progression-free survival estimates were 83.5% and 100%, respectively. In terms of toxicities, 71% of the surgical group had grade II–V toxicities as compared with 67% of patients in the RT arm. Average MDADI scores one year post-treatment were similar between arms and no one required a feeding tube at one year. Overall, this trial demonstrated a mortality risk with the upfront surgery approach but demonstrated that this lowered dose of RT had both positive oncologic outcomes and toxicity profile. This data was drawn from a recently presented abstract as publication is pending.

## 4. Surgery and Adjuvant Low-Dose Radiotherapy Approach

There are a number of studies that have investigated the role of upfront surgery with de-escalation of adjuvant RT. Those that are completed and those ongoing are discussed further.

The MC1273 trial (NCT01932697 (accessed on 1 January 2022)) was a single-arm phase II trial investigating whether post-operative RT dose reduction, from 60–66 Gy to 30–36 Gy, administered with weekly docetaxel, could reduce toxicity while maintaining both quality of life and high rates of disease-free survival [44,45]. Patients included in this study had HPV-associated OPSCC staged with the AJCC 7th edition as either stage III or IV with a smoking history of 10 pack-years or less. Those included underwent curative intent primary site surgical resection and neck dissection with negative margins and were subsequently stratified to one of two cohorts based on pathologic analysis. Group A had tumors with no extra-nodal extension (ENE) but had at least one other intermediate-risk factor (lymphovascular invasion (LVI), perineural invasion (PNI), involvement of ≥two regional lymph nodes, any lymph node > 3 cm in size, or ≥T3 primary tumor). Group B were ENE positive. The adjuvant therapy for group A (*n* = 37) consisted of 30 Gy RT and two cycles of docetaxel, while group B (*n* = 43) received 36 Gy RT and two cycles of docetaxel; 95% of the patients underwent transoral surgery and all completed their treatment plans. The average follow-up during this study was 35 months. In Group A, one patient had a distant recurrence at 12 months and Group B had nine patients with disease recurrence (three local, one regional, and five distant metastases to either lung or bone). The three patients with local recurrence all required revision margin excision intra-operatively after frozen sections were positive for disease. For the whole cohort, two-year distant metastasis-free survival was 94.9%, progression-free survival was 91.1%, and overall survival was 98.7% (the three deaths were secondary to cardiac or pulmonary causes, not due to their cancer). This study attempted to decrease the RT to a lower dose than other trials while using concurrent adjuvant docetaxel to make the effective dose higher, and still demonstrated locoregional control, progression-free survival, and overall survival rates similar to standard adjuvant therapy [46]. While Group B had a higher rate of negative disease outcomes (21% recurrence needing salvage), this was to be expected based on the ENE found in their disease. This study concluded that this aggressive RT de-intensification achieved similar results as historical controls. The toxicity of this treatment regimen was improved in early and late adverse events as compared with historical controls. Only one patient required percutaneous feeding supplementation which was removed one month post-treatment. Using patient reported outcome measures, pre-RT quality of life metrics were improved at one-year follow-up. It can be noted that intermediate-risk patients in this cohort, those with completely resected disease, between one to four positive lymph nodes, and without ENE, had a very good prognosis with this de-escalated regimen. Worse prognostic factors for progression were larger primary tumor size, greater than four positive lymph nodes, and ENE. These patients were at a greater risk of distant failures. One hypothesis may be that these high-risk patients require systemic therapy escalation to aid in treatment of micro-metastases for possible distant recurrence.

The AVOID phase II trial (NCT02159703 (accessed on 1 January 2022)) assessed patients with resected pT1–2, pN1–3, M0 HPV-related OPSCC, staged with the AJCC 7th edition, with no primary site risk factors and withheld RT (IMRT or IMPT) to the primary site to improve the toxicity profile [47]. All patients (*n* = 60) underwent TORS with neck dissection and were included if there were clean surgical margins >2 mm, no PNI, and no LVI. RT was not given to the primary tumor site, and only the involved neck was treated with 60–66 Gy, and the uninvolved neck with 54 Gy. Patients with ENE (*n* = 13) were treated with adjuvant CRT (nine weekly low-dose cisplatin, two high-dose cisplatin, two cetuximab). In this 60-patient cohort with an average follow-up of 2.4 years, only one patient had primary site recurrence, one patient developed regional neck recurrence, and two patients later presented with distant metastases. The locoregional recurrence patients underwent salvage surgical resection. The two-year local control rate was 98.3% and the overall survival was 100%. No patients required long-term percutaneous feeding tubes, but two patients required post-treatment feeding tubes which were later removed. Two patients had soft tissue necrosis and they had higher RT dose to the primary site than those without soft tissue necrosis (45.8 Gy versus 36.6 Gy). This was treated conservatively in both, and one of the patients used hyperbaric oxygen therapy. With this technique, the average RT dose to the primary site was 36.9 Gy, which was significantly lower than post-operative standard 60–66 Gy. This cohort demonstrated a good safety profile for risk-stratified de-intensified postoperative RT that aimed to avoid the primary resected site.

E3311 (NCT01898494 (accessed on 1 January 2022)) is a multi-institutional phase II trial that assessed the feasibility of reducing the dose of adjuvant RT in patients who underwent transoral surgery [48]. Patients included had stage T1–2, N1–2b HPV-associated OPSCC, as per AJCC 7th edition, and underwent TORS or TLM. In total, 359 patients were assigned one of four adjuvant treatment arms based on their post-operative pathological risk. Group A consisted of low-risk patients (*n* = 38), including those with T1–2 disease with negative margins >3 mm and N0–1 without ENE, and were given no adjuvant therapy. Group D patients were high-risk patients (*n* = 113) due to positive margins, >1 mm of ENE, or five or more positive lymph nodes, and received post-operative chemoradiation therapy. The population of interest of this trial were the intermediate-risk patients (Group B and C). These patients were defined as T1–2 primary tumors with negative margins or margins < 3 mm, N1–2 with ≤1 mm ENE, or up to four positive lymph nodes. Post-operatively, this group was randomized to receive adjuvant RT at either a reduced dose of 50 Gy (group B, *n* = 100), or a standard dose of 60 Gy (group C, *n* = 108). After approximately 35 months of surveillance, there was no significant difference in progression-free survival between groups. Two-year progression-free survival was 96.9% for arm A, 94.9% for arm B (50 Gy), 96.0% for arm C (60 Gy), and 90.7% for arm D. There were 16 deaths in the patient cohort (one in A, two in B, six in C, and seven in D). The two-year overall survival was 100% for arm A, 99.0% for arm B, 98.1% for arm C, and 96.3% for arm D. This trial evaluated treatment-related toxicities and noted a significantly different rate of grade III to V treatment toxicities between arms B and C (14% versus 24%, *p* = 0.03). E3311 utilized 50 Gy as the de-escalated RT dosage, which is still above the dosage tolerated by salivary glands and would be unlikely to improve this adverse event. Deasy et al. reviewed the effect of dose volume on salivary gland function and determined that severe xerostomia can be avoided at lower doses than what patients received in this trial [20]. Specifically, they determined that ideal doses to parotid glands are less than 25 Gy if both parotid glands or less than 20 Gy in at least one of the parotid glands. Another study determined that 39 Gy was the threshold dose for submandibular glands, where gland function may improve gradually over the two years post-RT if this level was not surpassed [49]. A similar study reported the effect of RT on the parotid glands and identified a threshold level of 26 Gy [50]. While it was not a primary endpoint, functional outcomes measured with the MDADI and Functional Assessment of Cancer Therapy-Head and Neck (FACT-H&N) for both intermediate-risk groups were similar in the E3311 study. This study concluded that a reduced dosage of adjuvant RT was an appropriate therapeutic option when pathological analysis identified intermediate-risk disease due to the progression-free survival, overall survival, and patient reported quality of life measures.

The SIRS phase II trial (NCT02072148 (accessed on 1 January 2022)) risk stratified HPV-associated OPSCC patients after pathological staging post-transoral surgery and neck dissection with AJCC 7th edition staging to receive different adjuvant treatments [51]. Low-risk patients were staged as pT1–2, pN0–2b, and were observed post-operatively. Intermediate-risk patients were those staged as pT1–2, pN0–2b with negative margins, with LVI and/or PNI, ≤3 lymph nodes (LNs), and <1 mm ENE and received 50 Gy adjuvant IMRT. High-risk patients had significant adverse features (>3 LNs, supraclavicular LNs, contralateral LNs, positive surgical margins, >1 mm ENE, or matted LNs), and therefore, received concurrent cisplatin and 56 Gy RT. There were 75 patients enrolled with 21 withdrawals. Overall, 54 patients were evaluated but 1 patient did not complete RT and was excluded from analysis; 24 patients were in the surveillance group (low risk), 14 patients received RT alone (intermediate risk), and 15 patients received CRT (high risk). Median follow-up was 43 months. Progression-free survival probability was 91.3% for Group 1, 86.7% for Group 2, and 93.3% for Group 3. The Global MDADI QOL scores improved with time and returned to baseline scores. No patients required long-term gastrostomy tube feeding. This trial demonstrated positive outcomes following post-operative risk stratification-based adjuvant treatment allocation in HPV-related OPSCC patients.

### Trials without Published Results

The PATHOS trial (NCT02215265 (accessed on 1 January 2022)) is similar to E3311, where T1–3, N0–2b, and M0 HPV-related OPSCC patients undergo minimally invasive transoral surgery and neck dissection with risk stratification based on pathological factors [48,52]. Low-risk patients are observed, intermediate-risk patients are randomized to 50 or 60 Gy RT (just as in E3311), and high-risk patients are randomized to adjuvant 60 Gy RT or 60 Gy RT with concurrent cisplatin.

The ADEPT phase III trial (NCT01687413 (accessed on 1 January 2022)) included T1–4a HPV-related OPSCC who underwent transoral surgery and neck dissection. Patients that were found to be ENE positive on pathological analysis were randomized to 60 Gy IMRT alone or with concurrent weekly cisplatin. The study was terminated due to funding issues and slow accrual in 2020 without publication but preliminary results are available on clinicaltrials.gov.

The Minimalist (MINT) trial (NCT03621696 (accessed on 1 January 2022)) is a phase II study of stage I–III resectable HPV-related OPSCC, staged with the AJCC 8th edition, in which patients undergo transoral surgery and neck dissection with adjuvant therapy determined by pathological risk. Low-risk patients receive 42 Gy RT, intermediate-risk patients receive 42 Gy RT and one dose of cisplatin, and high-risk patients undergo 60 Gy RT with concurrent cisplatin. There are no published results, but preliminary data are available on clinicaltrials.gov (accessed on 1 January 2022).

DART-HPV (NCT02908477 (accessed on 1 January 2022)) is a follow-up study from MC1273 where patients with resectable T1–3, N0–3, M0 HPV-associated OPSCC are randomized to standard CRT (60 Gy with concurrent cisplatin) versus 30–36 Gy with concurrent docetaxel, the regimen proposed from MC1273.

ADAPT (NCT03875716 (accessed on 1 January 2022)), the phase II trial, utilizes pathologic analysis post transoral surgery and neck dissection to plan adjuvant therapy. Low-risk patients are observed, intermediate-risk patients receive reduced dose RT (46 Gy), and high-risk patients receive adjuvant standard dose RT (60 Gy) without chemotherapy.

The DELPHI phase I trial (NCT03396718 (accessed on 1 January 2022)) includes patients with OPSCC who underwent primary site surgery and neck dissection with indications for adjuvant therapy. Patients with HPV-associated OPSCC are given one of three options of RT dosage with or without chemotherapy as needed and as determined by tumor board discussion and pathological analysis. The three RT dosage options are standard (60/66 Gy), reduced level 1 (54/59.4 Gy), and reduced level II (48.8/55 Gy). Patients with high-risk features were differentiated from those with intermediate-risk characteristics and were treated with the higher dose RT and concurrent chemotherapy.

NCT03729518 (accessed on 1 January 2022) is a phase II study from the Abramson Cancer Center of the University of Pennsylvania currently recruiting patients with pT0–3, N0–2b, M0 HPV-associated OPSCC, staged with the AJCC 7th edition, who have undergone TORS primary site resection and ipsilateral neck dissection. These patients will receive reduced-dose RT (IMRT or IMPT at 50 Gy to ipsilateral high risk neck and 45 Gy to contralateral side). Patients will receive adjuvant chemotherapy as well if criteria are met.

NCT02784288 (accessed on 1 January 2022), a phase II trial from the University of Michigan Rogel Cancer Center, recruited 34 patients who have potentially resectable T1–3, N0–2c, M0 HPV-related OPSCC [53]. The patients underwent up-front neck dissection and used the neck lymphadenopathy pathology results to guide treatment: low-risk patients (single lymph node < 6 cm, no ENE, no PNI, no LVI), will undergo transoral surgery of the primary cancer; intermediate-risk patients (≥2 LNs, without adverse features or a single node with LVI or PNI) will undergo RT; and high-risk patients (ENE positive) will complete transoral surgery and chemoradiation. While not yet fully published, this group explained their preliminary methodology in a parallel study within this same cohort. The preliminary clinical trial methodology is described in their publication on their goal of quantifying the circulating tumor DNA (HPV ctDNA) from plasma in HPV-associated OPSCC patients. This HPV ctDNA will be analyzed within this cohort to assess clearance of HPV ctDNA post-treatment and follow ctDNA for recurrence.

## 5. Altered Regimen of Chemoradiotherapy Approach

Concurrent CRT is the current standard of care, but is associated with significant side effects. There are studies completed and ongoing that have discussed de-intensifying the chemotherapy and/or the radiation regimen to decrease toxic effects. These are discussed below.

The NRG HN002 (NCT02254278 (accessed on 1 January 2022)) phase II trial included patients with T1–2 N1–2b M0, or T3 N0–2b M0 HPV-associated OPSCC (staging with AJCC 7th edition) and assigned them to concurrent 60 Gy IMRT (over 6 weeks) with cisplatin versus 60 Gy (over 5 weeks) alone [54]. This study of reduced IMRT dosage enrolled 306 patients, 157 patients were randomly assigned to concurrent chemoradiotherapy and 149 patients to IMRT alone group. The primary endpoint was progression-free survival at two years and swallowing quality of life at one-year via the MDADI patient reported outcome measure. Overall, seven patients did not complete the IMRT doses as per the assigned protocol (five patients refused, two received alternative therapy). In the cohort assigned to receive concurrent chemotherapy, five patients did not receive any and 45 patients terminated the regimen early. The median follow-up was 2.6 years, and 292 patients were followed for at least two years. The estimated two-year locoregional failure rates were 3.3% and 9.5% in the concurrent versus IMRT alone group, with a significant difference between arms. The overall-survival rates at two years were similar, 96.7% for the CRT group versus 97.3% for the IMRT group. The combination therapy group had higher rates of acute adverse events, however, the MDADI scores at one year were not significantly different from baseline (*p* = 0.78). The IMRT-alone group did not meet acceptability criteria as the progression-free survival rate was just 87.6%. For the chemoradiotherapy cohort, the two-year progression-free survival met acceptability criteria at 90.5%. This treatment arm met the predefined endpoints allowing development into a phase III trial.

NCT00606294 (accessed on 1 January 2022) is a pilot study from the Memorial Sloan Kettering attempting to de-escalate RT in concurrent CRT treatment for patients with T1–2, N1–2b (AJCC 7th edition) with HPV-related OPSCC after assessment of tumor hypoxia with fluorine-18-labeled fluoromisonidazole PET (^18^F-FMISO PET) [55]. It has previously been demonstrated that hypoxia mediates radiation resistance and is a negative prognostic factor for malignancies [56]. Therefore, in patients with no hypoxia on this nuclear imaging, treatment would consist of chemotherapy and de-intensification of RT to 30 Gy. Patients first underwent primary tumor resection and patients were included even if there were positive margins. Two to four weeks post-operatively, they had a fluorodeoxyglucose PET or computed tomography-based simulation for planning of RT, as well as the ^18^F-FMISO PET to evaluate pretreatment hypoxia status. Those without hypoxia initially or on pretreatment scanning would receive 30 Gy RT, with two cycles of cisplatin. If there was hypoxia on this first scan, patients were re-assessed approximately one week after starting chemotherapy to determine if there was an intra-treatment change and a possibility for RT dose de-escalation down to 30 Gy. The patients with persistent hypoxia on intra-treatment ^18^F-FMISO PET were treated with 70 Gy and two cycles of cisplatin. Patients underwent weekly MRIs post-treatment, and neck dissection at four months after CRT to assess pathological response. There were 18 patients who were included and analyzed in this study. Within this cohort, 6 patients had no evidence of hypoxia and 12 patients had pretreatment hypoxia. These six patients received 30 Gy RT with cisplatin. The 12 patients with hypoxia started the RT and cisplatin and at the intra-treatment scan follow up, nine patients had no further evidence of hypoxia. Therefore, these nine patients also received 30 Gy RT and cisplatin, however, one of the fifteen patients only received one dose of cisplatin. The median follow-up was 34 months. Eleven of these 15 patients had a complete pathological response on post-treatment neck dissection, two patients had minimal foci of residual disease with uncertain viability (without further treatment), and one patient had clinically significant residual disease with tumor regrowth seen on post-treatment MRI. The patient who did not receive his second cycle of cisplatin developed progressive locoregional disease. There were no grade III radiation-related toxicities observed in the de-escalated group. Two-year locoregional control, progression-free survival, and overall survival for the de-escalated cohort per protocol were 100%, 92.9%, and 92.9%, respectively. This pilot study has concluded that using hypoxia as a marker for radiosensitivity and de-escalation using this data is safe in HPV-related OPSCC. NCT03323463 is a phase II clinical trial currently recruiting patients within the same research group to assess this protocol without mandatory post-CRT neck dissection.

LCCC1120 (NCT01530997 (accessed on 1 January 2022)) is a completed phase II trial of patients with T0–3, N0–2c, M0 HPV-associated OPSCC (7th edition) who underwent concurrent CRT at de-escalated doses [57]. Patients received a six-week course of IMRT at 60 Gy with six weekly low doses of cisplatin (30 mg/m^2^), as opposed to standard 70 Gy and three cycles of high-dose cisplatin (100 mg/m^2^). Clinical and radiologic responses were assessed post-completion of CRT. If there was a complete clinical response at the primary site, patients underwent evaluation under anesthesia and primary site directed biopsies. In cases of partial primary site response post-CRT, transoral surgery was performed to resect the remaining disease. Finally, any non-N0 patient on clinical or radiologic exam underwent selective neck dissection (SND), to remove at least all previously involved nodal levels [58]. The primary endpoint of this trial was pathologic complete response (CR), using the benchmark value of 87% locoregional control as this is the quoted three-year locoregional control rate with standard dosing CRT [8]. There were 45 patients recruited and 43 patients completed the planned protocol and were included in the analysis. At a median 14-month follow-up from onset of treatment, there was no measurable tumor present on physical and radiologic examination in 64% of the patients. Post-operatively, the pathological CR rate was 86% (37/43). There were 17 patients who required a feeding tube for an average of 15 weeks, but no patient required long-term supplemental feeding. This was a great improvement as compared with the PARADIGM study, where 85% of head and neck cancer patients receiving two cycles of high-dose cisplatin and 72 Gy RT required feeding tube placement [59]. In 2018, this group published updated results of their cohort after a median 36-month follow-up, reporting a three-year locoregional control, distant metastasis-free survival, and overall survival rates were 100%, 100%, and 95%, respectively [60]. The six patients with incomplete pathological response were all alive with no evidence of disease. At three years post-treatment, this cohort’s global quality of life returned to baseline and patients did not suffer from significant swallowing dysfunction. This favorable toxicity profile even noted a continued improvement of the acute onset xerostomia that peaked in patients at around six to eight weeks. This group has been working towards identifying the optimal CRT de-escalation regimen, and therefore moved, forward with LCCC1413.

LCCC1413 (NCT02281955 (accessed on 1 January 2022)) is a follow-up to NCT01530997 (study above) and utilized the same inclusion criteria [61]. The same de-escalated treatments were used but the protocol did not necessitate post-treatment surgical evaluation. Instead, they used positron emission tomography-computed tomography (PET-CT) to assess need for surgical evaluation. All patients received 60 Gy IMRT (*n* = 114), 80% of the patients staged to receive chemotherapy completed at least four cycles of cisplatin, and 11% of patients received cetuximab upfront due to contraindications to cisplatin. The median follow-up was 31 months and 81% of patients were followed for at least two years. The post-treatment complete response on PET-CT was 93% (*n* = 8 residual disease) at the primary site and 80% in the neck. Of the eight patients with residual disease at the primary site on imaging, six patients were observed with no local recurrence at two years, two patients were biopsied, and one patient had local persistent disease and died. Of eleven patients who had a neck dissection for residual neck disease, four patients had pathological residual disease. All are alive and with no evidence of disease at follow-up. One patient died of neutropenic sepsis. Two-year locoregional control, progression-free survival, and overall survival were as follows: 95%, 86%, and 95%, respectively. Additionally, 38 of 113 patients required a feeding tube for a median duration of 10.5 weeks, but none were permanent. Patient-reported outcome measures demonstrated decreases in quality of life and a higher symptom burden after completion of treatment, but all returned to baseline after 6 months.

### Trials without Published Results

LCCC1612 (NCT03077243 (accessed on 1 January 2022)) is a follow-up study to LCCC1120 and LCCC1413 after learning that the de-escalation regimen is efficacious in these two studies (6-week course of IMRT at 60 Gy with 6 weekly low doses of cisplatin (30 mg/m^2^), Within this trial, they will identify smoking history and p53 mutational status. Patients with a significant smoking history who are wild-type p53 will be de-escalated, however, those with mutated p53 will not receive the de-escalated therapy. The goal of this trial is to identify who can safely be de-escalated.

NCT01088802 (accessed on 1 January 2022) is a phase II clinical trial from the Sidney Kimmel Comprehensive Cancer Center at Johns Hopkins consisting of HPV-associated T1–3, N0–2c, M0 OPSCC reducing IMRT dosage from 70 Gy to 63 Gy with concurrent cisplatin therapy.

EVADER (NCT03822897 (accessed on 1 January 2022)) is a phase II clinical trial of HPV-associated OPSCC AJCC 8th edition T1–3, N0–1 investigating an experimental RT with altered RT volume for the neck. This study is assessing whether omitting RT from specific low-risk lymph node areas is safe and efficacious. The two groups will both receive experimental RT, but one will also have standard cisplatin chemotherapy while the other will not.

## 6. Targeted Therapy with EGFR Inhibitor versus Cisplatin Approach

The trials that have investigated targeted therapy with the monoclonal anti-EGFR antibody cetuximab as a potential replacement to standard cisplatin-based chemotherapy are discussed below.

RTOG1016 (NCT01302834 (accessed on 1 January 2022)) was a phase III randomized, prospective clinical trial exclusive to patients diagnosed with T1–2, N2a–3 or T3–4, N0–3 HPV-positive OPC (AJCC 7th edition staging) investigating whether replacing cisplatin with cetuximab would maintain high efficacy and reduce toxicities [62]. Cetuximab, an epidermal growth factor receptor inhibitor, was proposed as previous studies comparing IMRT alone with IMRT and cetuximab in patients with locally advanced head and neck cancer had improved control and mortality rates without greater toxicity burden [63]. All patients in the RTOG1016 trial received standard 70 Gy IMRT over six weeks and were randomized to weekly cetuximab (*n* = 399) or two cycles of high-dose cisplatin on Days 1 and 22 of radiotherapy (*n* = 406). The median follow-up was 4.5 years and there were 133 deaths recorded; 78 and 55 patients died in the cetuximab and cisplatin groups, respectively. The rate of grade III–IV acute adverse events was similar between the two groups, but the side effect profiles were different with rash being more common with cetuximab and myelosuppression, kidney injury, and hearing impairment occurring more commonly with cisplatin. Progression-free survival was significantly decreased in the cetuximab group (67.3 vs. 78.4%, *p* = 0.0002). Most importantly, when assessing the cohort for overall survival outcomes, radiotherapy and cetuximab did not meet the criteria for non-inferiority as compared with cisplatin. Therefore, cetuximab is not an appropriate substitute for cisplatin for patients with HPV-related OPSCC.

The De-ESCALaTE HPV trial (NCT01874171 (accessed on 1 January 2022)) was another phase III clinical trial that compared cetuximab to cisplatin chemotherapy in patients with T3–4, N0 or T1–4, N1–3 HPV-related OPSCC staged with AJCC 7th edition [64]. Patients received 70 Gy radiotherapy and were randomized to concurrent seven weekly doses of cetuximab (*n* = 152) or three cycles of high-dose cisplatin on Days 1, 22, and 43 of radiotherapy (*n* = 152). The results were similar to *ROTG1016* where the overall mean number of toxicity events was similar between the two cohorts. However, there was a significant difference in two-year overall survival of 97.5% for cisplatin versus 89.4% for cetuximab, *p* = 0·001, and two-year recurrence rate of 6.0% for cisplatin versus 16.1% for cetuximab, *p* = 0·0007. Therefore, this study also concluded that cetuximab is inferior to cisplatin and should not be used in these patients.

TROG12.01 (NCT01855451 (accessed on 1 January 2022)) investigated cetuximab to weekly cisplatin chemotherapy [65]. Using the AJCC 7th edition, patients with HPV-associated OPSCC stage III (except T1–2, N1) or stage IV (except T3, N3, or M1) with less than 10 pack-year smoking history, or if greater than 10 pack-year smoking exposure must be N0–2a were included. Patients received standard-dose 70 Gy RT and were randomized to receive concurrent cetuximab (*n* = 90) or weekly cisplatin (*n* = 92). The symptom severity was similar between the two groups. However, the three-year failure-free survival rates were 80% in the cetuximab arm and 93% in the cisplatin group (hazard ratio 3, *p* = 0.015). Therefore, cisplatin remains superior to cetuximab as the standard of care in non-surgical management of this disease.

### Trial without Published Results

NRG HN005 (NCT03952585 (accessed on 1 January 2022)) is a phase II/III trial of T1–2, N1, M0, or T3, N0–1, M0 HPV-associated OPSCC (AJCC 8th edition). Patients are randomized to one of three arms: (1) concurrent CRT with two cycles of cisplatin and 70 Gy IMRT, (2) two cycles of cisplatin with concurrent 60 Gy IMRT, or (3) two cycles of cisplatin 60 Gy IMRT with the addition of nivolumab. Patients can receive IMRT or image-guided radiotherapy. This study is assessing whether reduced dose RT with nivolumab is as efficacious as standard dose radiation therapy and cisplatin in this cohort.

## 7. Neoadjuvant Chemo with Consolidation Surgery Approach

The NeCTORS (NCT02760667 (accessed on 1 January 2022)) phase II clinical trial utilized neoadjuvant chemotherapy and TORS to de-escalate the treatment of stage III and IVa (AJCC-7) HPV-associated OPSCC, reserving radiotherapy for salvage. It was based on the efficacy of the approach shown previously [29,30,66]. The neoadjuvant chemotherapy approach has been shown to be highly effective to downstage the cancer and decrease the tumoral burden in the neck and the primary site to allow definitive surgical consolidation of treatment with negative margins without adjuvant RT/CRT. It also has the added benefit of providing systemic treatment to prevent metastatic spread of disease, which is a concern in patients with advanced neck disease and accounts for half of the mortalities despite locoregional control with standard CRT. The approach combines systemic escalation with more radical locoregional de-escalation on the premise that most of the late toxicity of the treatment of OPC comes from locoregional adverse therapy effects. The surgical margin of the primary tumor is immediately outside the pre-chemotherapy tumor margin. Mucosal margins of the primary tumor are tattooed, when it extends outside of the tonsillar fossa or base of tongue (BOT), before starting chemotherapy in order to map out the subsequent surgical resection. Patients undergo three cycles of neoadjuvant chemotherapy with cisplatin and docetaxel, and then TORS with SND is performed. The SND is unilateral for tonsillar fossa cancers, unless they extend into the BOT or the soft palate beyond 1 cm. This trial aimed to avoid RT to the head and neck altogether, which is thought to be the main driver of post-treatment morbidity in OPSCC patients. In a prior case series based on NeCTORS, a cohort of 55 patients (T1–2, N1–2c, T3N0–2c, with any number of nodes, AJCC 7th edition) were compared to a propensity T and N-matched cohort from a historical control of 142 patients who underwent concurrent CRT. In the NeCTORS group only 2/55 patients required adjuvant CRT due to unresectable positive margins following TORS, and none required salvage RT for recurrence. The five-year disease-free survival was 96.1% in trial participants and 67.6% in the historical CRT controls. There were seven (12.7%) severe toxicity events without permanent sequelae in the neoadjuvant chemotherapy and surgery group as compared with 35 (24.6%) events in the control group. While a nasogastric feeding tube was inserted for immediate post-operative nutritional support for a median duration of six days, no patients required gastrostomy tube placement in the NeCTORS group, as opposed to 24.5% of the control group who remained gastrostomy tube dependent at 12 months post-treatment. Distant metastases are the main reason for failure of HPV-associated OPSCC post-treatment [67,68]. Given the proven efficacy of the neoadjuvant chemotherapy and no patient developing distant metastases in prior studies, it is believed that this is an effective method against undetectable possible micro-metastases [30]. The aim of this trial was to change the customary treatment from radiation-based, to surgery-based approaches in hopes of limiting chronic RT-based adverse events. RT was reserved for pathologic adverse findings including >three nodes with persistent tumor, ENE > 2 mm, positive margins, as salvage for recurrences, and for management of second primaries.

## 8. Neoadjuvant Chemotherapy and Low-Dose Radiotherapy Approach

The next studies investigate induction chemotherapy and low-dose adjuvant RT. These completed studies are described below.

The E1308 (NCT01084083 (accessed on 1 January 2022)) phase II clinical trial aimed to study neoadjuvant chemotherapy and reduced-dose IMRT with cetuximab in stage III and IV HPV-associated OPSCC (7th edition staging) [69]. Enrolled patients (*n* = 80) underwent induction chemotherapy with three cycles of cisplatin, paclitaxel, and loading dose followed by weekly cetuximab and concurrent IMRT. Radiological (CT or MRI) and clinical reassessment was performed within 14 days of induction chemotherapy. In cases of complete response, patients were treated with reduced-dose IMRT (54 Gy in 27 fractions), while cases of partial or no response received a standard 69 Gy in 33 fractions. Fifty-six (70%) of the enrolled patients had a complete clinical response, and 51 patients underwent reduced-dose IMRT and weekly cetuximab (five protocol deviations treated with 69 Gy); 18 patients had incomplete response to induction chemotherapy, 10 patients proceeded with 69 Gy IMRT, and 8 patients with protocol deviations were treated with 54 Gy. With regards to grade III toxicity, the cohort receiving 54 Gy of RT and concurrent cetuximab, and the most frequent adverse events experienced were mucositis (30%) and dysphagia (15%). In the 69 Gy RT arm, there were higher rates of these same adverse events, with 47% of the patients suffering from mucositis and 29% of the patients having dysphagia. The two-year progression-free survival and overall survival for the 51 patients with complete clinical response receiving 54 Gy RT was 80% and 94%, respectively. All patients with treatment failures within two years had a greater than 10 pack-year smoking history. In a post-hoc analysis including only those with ≤10 pack-year smoking history, <T4, and <N2c, the two-year progression-free survival and overall survival increased to 96% and 96%, respectively.

The Quarterback trial (NCT01706939 (accessed on 1 January 2022)) was a phase III clinical trial of HPV-related stage III or IV OSPCC without distant metastases, staged with the 7th edition [70]. Patients (*n* = 22) received three cycles of docetaxel, cisplatin, and fluorouracil. Patients were evaluated post-induction chemotherapy and if there was partial or complete response, they were randomized to standard CRT (*n* = 8, 70 Gy) or reduced CRT (*n* = 12, 56 Gy), with carboplatin. The goal of the RT regimen is to lower the mean dose to the parotids to under 26 Gy and under 50 Gy to the pharyngeal constrictors, when possible. When evaluating the primary site of the whole cohort after induction chemotherapy, 16 patients had complete responses and four patients had partial responses. At the nodal basin, 16 patients had complete responses, three patients had partial responses, and one patients was unable to be evaluated. After CRT, all patients had primary site complete clinical and radiologic responses, and 19/20 had neck complete responses. The one remaining patient underwent a salvage neck dissection for residual disease. The median follow-up period was 56 months. The three-year progression-free and overall survival rates were 87.5% for standard CRT (7/8) and 83.3% for reduced CRT (10/12 patients) (same rates for both endpoints). Non-inferiority of reduced CRT dosages could not be demonstrated given the limited number of enrolled participants. However, it should be noted that there was clinical response in all participants and all treatment failures were within four months of treatment completion, with no further recurrences in long-term follow-up. The Quarterback II trial (NCT02945631 (accessed on 1 January 2022)) is a follow-up trial to the aforementioned study, also testing RT dose reduction (56 Gy) after induction chemotherapy (docetaxel, cisplatin, 5-fluorouracil) and is currently recruiting.

The OPTIMA trial (NCT02258659 (accessed on 1 January 2022)) was a phase II clinical trial of patients with HPV-associated T1–4, N2–3, M0, or T3–4, any N, M0 OPSCC (AJCC 7th edition). Enrolled patients were first risk stratified based on baseline characteristics as low-risk patients (≤T3, ≤N2B, ≤10 pack-year smoking history) or high-risk patients (T4 or ≥N2C or >10 pack-year smoking history), and all were treated with induction chemotherapy (three cycles of carboplatin and nab-paclitaxel) [71]. Baseline risk status and response to induction chemotherapy then guided therapy: (1) low-risk patients with >50% response received 50 Gy RT alone (RT50), (2) low-risk patients with 30–50% response and high-risk patients with >50% response received 45 Gy CRT (CRT45), and (3) patients with lesser response received standard-of-care 75 Gy CRT (CRT75). Given the significantly reduced RT/CRT doses in the experimental arms, patients underwent surgical evaluation four to six weeks post completion of RT/CRT via modified/selective ND and possible biopsy/excision of the primary as deemed appropriate by the surgeon for pathologic confirmation of response. There were 62 patients enrolled and there was a median follow-up of 29 months. There were 28 low-risk and 34 were high-risk patients. The response rate following induction chemotherapy for the whole cohort was 89%, with 71% of the patients experiencing greater than 50% tumor size reduction. In the low-risk cohort, 20/28 patients received RT50, 6/28 patients received CRT45, and 2/28 patients received CRT75. In the high-risk group, 24/34 patients received CRT45, 9/34 patients received CRT75, and one patient transferred care. The pathological complete response rate for all patients (*n* = 52, 19 RT50, 28 CRT45, and 5 CRT75) in whom post-treatment surgery was performed as per protocol was 90% (47/52): 92% (43/47) for patients receiving de-escalated treatment arms and 80% (4/5) for the poor responders treated with standard CRT. Two-year progression-free survival and overall survival rates were 95% and 100% for low-risk patients, and 94% and 97% for high-risk patients, respectively. PEG-tube requirement at 12 months post-treatment were 0/28 in the low-risk group of patients and 2/34 in the high-risk patients. This trial concluded that there is a good pathological and toxicity-related result to induction chemotherapy and risk-stratification modifications of adjuvant RT or CRT.

OPTIMA-II (NCT03107182 (accessed on 1 January 2022)) is a follow-up phase II trial for HPV-positive OPSCC aiming to determine radiologic response to induction chemotherapy and additional induction nivolumab. Patients will receive induction carboplatin, nab-paclitaxel, and nivolumab. Treatment groups will be stratified based on staging and pathological features, as well as volume reduction from the induction chemotherapy. Options include TORS or radiation with or without chemotherapy. OPTIMA-II utilizes the same risk stratification regime as the OPTIMA trial, but low-risk patients with >50% response will be offered either TORS and SND as a definitive treatment if technically feasible with adjuvant radiation for adverse pathologic features, or the same 50 Gy RT as in the above study. Low-risk patients with a tumor volume response between 30 and 50% or high-risk patients with >50% reduction will receive de-intensified chemoradiation (intermediate dose of 50 Gy). Low-risk patients with <30% reduction or high-risk disease with <50% reduction or any patients with progressive disease during induction chemotherapy will undergo standard chemoradiotherapy with 70–75 Gy and concurrent cisplatin or paclitaxel, 5-fluorouracil, and hydroxyurea. Adjuvant nivolumab will be offered to all patients for 6 months post completion of definitive therapy. This study is ongoing with no results available thus far.

The CCRO-022 (NCT02048020/NCT01716195 (accessed on 1 January 2022)) phase II multicentric trial included stage III or IV HPV-associated OPSCC [72]. Patients underwent induction chemotherapy of two cycles of paclitaxel and carboplatin and further chemotherapy and IMRT regimens were determined by the response [72]. Those with no response (*n* = 20) were treated with 60 Gy adjuvant IMRT, while those with partial or complete response (*n* = 24) were treated with 54 Gy. The primary objective of the study was to estimate the two-year progression-free survival. Three (7%) of 44 patients developed local-regional failure, two of whom had received 60 Gy. One patient within the 60 Gy group developed distant metastasis and underwent further systemic therapy and their disease remained stable at the time of publication. Overall, this study yielded survival results similar to historical controls treated with standard CRT as the two-year progression-free survival rate was 92%. There was also found to be a reduction in long-term side effects with the lower dose RT using standardized patient reported outcome measures.

## 9. Discussion

As evidenced by this review, the management of HPV-associated OPSCC is a topic of great interest given the favorable survival outcomes and the need to personalize the treatment and improve the patient-reported quality of life and functional outcomes. Various treatment de-escalation approaches have been suggested to achieve optimal oncologic outcomes while minimizing treatment-associated morbidity. These include surgery and risk-based adjuvant treatment de-escalation, altered regimen CRT, neoadjuvant chemotherapy with surgical consolidation, and neoadjuvant chemotherapy with risk-based RT consolidation.

Trials exploring upfront surgery and employing pathology-based de-intensification, perhaps provide improved risk stratification and patient candidacy for de-escalated adjuvant treatment regimens. The largest study was E3311, where 359 patients were enrolled, and 113 patients had adjuvant CRT due to multiple positive lymph nodes or ENE. Almost a third (31%) of the enrolled participants having had upfront surgery required adjuvant CRT, thereby, received tri-modality therapy [48]. The major advantage of this approach was within the intermediate-risk category, which included those with two to four positive lymph nodes without ENE. In this cohort of 206 patients, it was determined that there was no difference in oncological outcomes and patients were subsequently treated with 50 Gy RT instead of 60 Gy. While this de-escalation is significant for post-treatment morbidity, 50 Gy is well beyond the salivary gland tolerance for RT and may lead to significant toxicity [20]. However, the results from the subsequent MC1273 trial described in the manuscript suggest upfront surgery with concurrent CRT may allow major RT dose de-escalation to 30–36 Gy [44]. While these patients would nonetheless receive tri-modality therapy, the reduced-dose adjuvant RT is more likely to spare salivary gland function.

The role of pathological ENE as a prognostic factor in HPV-associated OPSCC remains somewhat controversial. Indeed, in the updated AJCC 8th edition staging manual of HPV-related OPSCC, the pathological staging of lymphadenopathy, now, only relies on the number of positive lymph nodes, with no inclusion of ENE, nodal size, or laterality. However, concern has been raised within the head and neck oncology community that ENE may, in fact, impact survival and should continue to be a prognosticator for HPV-related OPSCC. A review of the National Cancer Database (NCDB) from 2010 to 2012 included 1043 patients with HPV-related OPSCC and examined the impact of ENE. ENE-positive patients had a worse three-year overall survival as compared with patients without ENE (89.3% vs. 93.6%, *p* = 0.01) [73]. In the ENE-positive cohort from this study, those receiving adjuvant concurrent CRT versus adjuvant RT alone did not have a difference in three-year overall survival (89.6% vs. 89.3%, *p* = 0.55). Therefore, it appears the addition of chemotherapy has a limited role in patients with ENE. A similar study revealed consistent results when analyzing the NCDB from 2010 to 2014. This study included 3745 patients with primary HPV-related OPSCC and 41% of node-positive cases demonstrated ENE [74]. ENE was more commonly found in pN2 (69.4%) disease as compared with pN1 (35.5%) and four-year overall survival was 92% in the ENE-negative patients, while just 85% in the ENE-positive cases (*p* < 0.001). These results remained significant when stratifying by nodal stage. Further research using this same database and expanding the population cohort to 2015 noted that ENE-negative patients had a higher five-year survival than ENE-positive patients (92.6% vs. 84.0%) and ENE-positivity was associated with a 1.90 hazard ratio of death [75]. Another study using the NCDB determined that ENE was a negative prognostic factor in both HPV-related and unrelated OPSCC, although with a worse overall survival in HPV-negative disease [76]. The upfront surgery approach allows pathologic identification of ENE and, as such, may allow better risk-based stratification to assess patient candidacy for de-escalation adjuvant regimens. Indeed, in patients with pathologic ENE, standard treatment should be favored over de-escalation, owing to the demonstrated 4–8% increased five-year mortality even with standard doses/volumes.

Our modern definitive CRT regimens have certainly greatly evolved in recent years. Docetaxel, cisplatin, and 5-fluorouracil have been used concomitantly with good results [77]. However, the use of 5-fluorouracil has been questioned given its additional toxicity profile. Indeed, in a retrospective study of patients undergoing chemotherapy for locally advanced head and neck cancer as compared with carboplatin plus 5-fluorouracil to cisplatin, tolerance was assessed by the percentage of patients completing the three cycles of chemotherapy. It was found that only 60.2% of patients receiving carboplatin with 5-fluorouracil completed three cycles, in contrast to 76.7% of patients treated with cisplatin. Therefore, head and neck oncologists have since largely abandoned routine triple agent chemotherapy [78]. There are many trials investigating CRT dose or volume de-escalation. NRG HN002 is a well-known study where positive findings have concluded that the RT dose in CRT could be lowered to 60 Gy from 70 Gy, while maintaining efficacy [54]. This study also demonstrated that the results were dependent on the concomitant chemotherapy, as the RT alone group did not meet the minimum progression-free survival 90% threshold for acceptability. While there is benefit from decreasing the RT dose by 10 Gy, it remains above the threshold of salivary gland tolerance, as previously mentioned [20]. It should be noted that this study was limited as it excluded radiographically matted lymph nodes, a common finding in OPSCC. In the Memorial Sloan Kettering approach employing functional imaging to assess hypoxia, the patients receiving significant RT dose de-escalation down to 30 Gy with concurrent cisplatin demonstrating excellent survival results [55]. Using hypoxia as a marker for radiosensitivity proved a promising avenue for individualized dose de-escalation, with a 60% RT dose reduction in 15/18 patients within the study, and no radiation-related grade III toxicities noted.

The next de-intensification approach for HPV-related OPSCC utilizes neoadjuvant chemotherapy to decrease the tumoral burden and allow definitive surgical resection without adjuvant RT. This approach allows radical de-escalation of the locoregional treatment to surgery only in the vast majority of patients with HPV-associated OPSCC, reserving RT for salvage treatment. The neoadjuvant chemotherapy de-escalates the surgery by decreasing gross tumoral bulk and nodal disease, allowing smaller resection just outside the pre-chemotherapy tumor margin as opposed to standard 1 cm margin, while potentially addressing distant micro-metastasis with induction systemic therapy [30]. Sparing of RT also allows complete avoidance of all minor and major salivary glands. This approach allows for saving RT for salvage, for unanticipated pathologic findings, for recurrence, and for potential future second primary cancers within the head and neck region. An important consideration with surgical management is the heightened risk of post-operative bleeding and its associated morbidity or mortality. Nonetheless, it is felt this risk is likely smaller than upfront surgery approaches where larger resection margins are typically required.

Similar to the above protocol are the trials investigating the efficacy of neoadjuvant chemotherapy with risk-based RT dose de-escalation. However, the lowest doses of RT given to patients in trials discussed in this category were 50 Gy RT or 45 Gy with concurrent chemotherapy in the OPTIMA study [71]. These decreased doses are a great improvement and will likely improve the side effect profile, but still carry a high-risk of radiation-induced xerostomia and dysphagia [20,49,50].

Targeted therapy with the monoclonal anti-EGFR antibody cetuximab has been investigated as a potential replacement to standard cisplatin-based chemotherapy, in hopes of further decreasing toxicity. However, two phase III studies (De-ESCALaTE HPV and RTOG1016) have concordantly reported inferiority in oncologic outcomes [62,64]. Subsequent publications have highlighted the controversial rationale of EGFR-targeting in HPV-positive OPSCC, as the pathogenesis of these tumors was largely related to viral oncoproteins E6 and E7 rather than the altered signaling pathways these agents target [79]. Thus, authors have postulated these early trials perhaps lacked a strong preclinical basis and highlight the need for intensive experimental studies preceding large clinical trial.

## 10. Conclusions

HPV-associated OPSCC is an evolving field due to the younger population and the markedly improved prognosis [8]. The longer lifespan means that these patients live for many years with the sequelae of treatments, and therefore, quality of life has become a priority. Specifically, emphasis must be placed on treatment modality optimization and selection due to the effects on patents’ senses, functional capabilities, and emotional expressions. These efforts to improve quality of life have been noted with great advancements in radiotherapy techniques, chemotherapy regimens, surgical approaches, and the use of personalized de-escalated combination therapies. Comparing these novel studies is difficult, and therefore, it is difficult to determine the best approach, due to a variety of reasons including differences in patient inclusion criteria such as staging and tumor characteristics, and center-specific factors such as equipment availability and provider expertise. It may well be that more therapeutic options that are equally effective will be available to this patient population and the choice will be driven by patients’ preferences for short- and long-term outlooks. In selecting the treatment, while five-years survival and outcomes are the norm for decision making, in this otherwise healthier and younger patient population, the long term sequela of treatment and outcomes over a 20–30 year outlook need be strongly considered. Improvements in our understanding of the biology of HPV-related disease have caused a shift towards more individualized approaches based on patients and tumor factors. The employment of the novel techniques discussed in this paper will hopefully maintain or improve current mortality rates, while significantly reducing the long-term morbidities in low-risk patients.

## Figures and Tables

**Table 1:** Overview of both published and ongoing treatment de-escalation clinical trials for human papillomavirus-associated oropharyngeal squamous cell carcinoma.

Study Name	NCT Code	Phase	Status	Eligibility	De-Escalation Strategy	Outcomes
**Upfront surgery and pathology-based adjuvant therapy**						
**ORATOR**	NCT01590355	II	Complete	T1–T2, N0–2 OPSCC (7th edition)	Patients randomized to: Surgical arm: TOS and ND ± adjuvant therapy (60 Gy RT or 64 Gy RT and chemotherapy) RT: 70 Gy ± high dose cisplatin (carboplatin or cetuximab if unfit)	Surgery group (*n* = 34): 16 patients received adjuvant RT, 8 patients received adjuvant CRT RT group (*n* = 34): 2 patients withdrew, 23 patients received concomitant CRT RT group had a better one-year swallowing-related quality of life, however, not a clinically meaningful difference ~4 Year follow-up
**ORATOR2**	NCT03210103	II	Complete, no published results	T1–2, N0–2 potentially resectable HPV-related OPSCC (8th edition)	Patients are risk stratified by smoking history, then randomized to de-intensified 60 Gy RT ± weekly cisplatin or TOS and ND ± adjuvant 50 Gy RT	Surgery group (*n* = 31), RT group (*n* = 30). Recruitment closed early due to two treatment related deaths in the surgical arm Two-year OS estimates were 89.1% in the TORS group and 100% in RT group The two-year PFS estimates were 83.5% in the TORS group and 100% in the RT group 71% Of the surgical group had grade 2–5 toxicities versus 67% of patients in the RT arm
**Surgery and de-escalation of adjuvant radiotherapy**						
**MC1273**	NCT01932697	II	Complete	Resectable HPV-related OPSCC, stage III or IV, ≤10 PY (7th edition)	All patients underwent surgery with curative intent. Post-operatively deemed high risk if ENE, LVI, PNI, ≥2 regional LN involved, any LN > 3 cm, or ≥T3 primary tumor. Stratified based on ENE: ENE negative: 30 Gy and docetaxel ENE positive: 36 Gy and docetaxel	Group A (*n* = 37) (1 distant recurrence) Group B (*n* = 43) (4 locoregional recurrence and 5 distant metastases) Whole cohort, two-year DMFS, PFS, and OS were 94.9%, 91.1%, and 98.7%, respectively This aggressive RT de-intensification achieved similar results as historical controls Toxicity and adverse events were improved as compared with historical controls Pre-RT QOL scores were improved at one year follow-up
**AVOID**	NCT02159703	II	Complete	Resectable pT1–2, pN1–3 HPV-related OPSCC (7th edition)	All patients undergo TORS and ND on with >2 mm margins, no PNI, no LVI. All patients receive adjuvant therapy to neck only (no primary site): Neck involved in disease: 60–66 Gy Neck uninvolved in disease: 54 Gy With concurrent chemotherapy if ENE+	All patients received adjuvant RT at 60–66 Gy (*n* = 60), ENE+ received concurrent CRT (*n* = 13) Follow up of 2.4 years Mean primary site radiation of 36.9 Gy Recurrence: primary site (*n* = 1), regional recurrence (*n* = 1), distant metastases (*n* = 2) Two-year LCR 98.3%, OS 100% at the time of analysis Adverse events: late soft tissue necrosis in the primary site with conservative management (*n* = 2) No long-term feeding tube dependence (*n* = 0)
**E3311**	NCT01898494	II	Complete	T1–2, N1–2b HPV-related OPSCC (7th edition)	All patients undergo TOS and ND. Post-operative risk stratification: Group A = Low risk = pT1–2, pN0–1 + negative margins: observation Intermediate risk = negative margins, ≤1 mm ENE, 2–4 LN involved, PNI or LVI: randomized to Group B 50 Gy adjuvant RT Group B 60 Gy adjuvant RT Group D high risk = positive margins, >1 mm ENE, >5 LN involved: 66 Gy adjuvant RT with concurrent cisplatin	Group A (*n* = 38), Group B (*n* = 100), Group C (*n* = 108), Group D (*n* = 131) Follow up period of 35 months No significant difference in PFS or OS: PFS 96.9% for arm A, 94.9% for arm B (50 Gy), 96.0% for arm C (60 Gy), and 90.7% for arm D OS was 100% for arm A, 99.0% for arm B, 98.1% for arm C, and 96.3% for arm D MDADI and FACT-H&N for both intermediate-risk groups were similar
**SIRS**	NCT02072148	II	Complete	T1, N1–2b or T2, N0–2b HPV-related OPSCC with <20 PY (7th edition)	All patients undergo TOS and ND. Post-operative risk stratification: Low risk = pT1–2, pN0–2b, no high risk features: observe Intermediate risk = pT1–2, pN0–2b, negative margins, LVI, PNI, <3 LNs, <1 mm ENE: 50 Gy adjuvant RT High risk ≥ 3LN, positive margins, ENE+, contralateral LNs: 56 Gy adjuvant RT with concurrent cisplatin	Group A (25), Group B (15), Group C (14) Median follow up 43.9 months PFS probability was 91.3% for Group 1, 86.7% for Group 2, and 93.3% for Group 3 Global MDADI QOL scores improved with time and returned to baseline scores
**PATHOS**	NCT02215265	II/III	Accrual	T1–3, N0–2b HPV-related OPSCC (7th edition)	All patients undergo TOS and ND. Post-operative risk stratification: Low risk = pT1–2, no adverse features: observe Intermediate risk = T1–3, N2a-b, PNI, LVI, 1–5 mm margins: randomized to adjuvant RT of 50 Gy or 60 Gy High risk = positive margins (<1 mm), >1 mm ENE: randomized to adjuvant 60 Gy RT or 60 Gy RT with concurrent cisplatin	N/A
**ADEPT**	NCT01687413	III	Accrual	Resectable T1–4a HPV-related OPSCC, ENE positive	All patients undergo TORS and ND, nodal disease with ENE randomized to 60 Gy RT alone or with concurrent weekly cisplatin	N/A
**MINT**	NCT03621696	II	Complete, no published results	Stage I-III resectable HPV-related OPSCC (8th edition)	All patients undergo TOS and ND. Post-operative risk stratification: Low risk = <T4, <cN3, no ENE, negative margins: 42 Gy adjuvant RT Intermediate risk = <T4, <cN3, ENE, or positive margins = 42 Gy adjuvant RT with one dose cisplatin High risk = T4, cN3: 60 Gy RT with concurrent cisplatin	Preliminary results available on clinicaltrials.gov
**DART-HPV (follow-up phase III randomized clinical trial to MC1273)**	NCT02908477	III	Complete, no published results	Resectable T1–3, N0–3, M0HPV-related OPSCC(7th edition)	Patients are randomized to: CRT with 60 Gy and cisplatin if high risk or Docetaxel with 30 Gy (36 Gy if high risk)	N/A
**ADAPT**	NCT03875716	II	Accrual	Resectable HPV-related OPSCC, T0–2, N0–1, M0 (8th edition)	All patients undergo TOS and ND. Post-operative risk stratification: Low risk = pT1–2, N0–1, minimum of 15 LNs examined, ≤2 LN involved, no ENE: observation Intermediate risk = pT1–2, N0–2, >2 LNs involved, <15 LNs examined, positive LNs in levels Ib, IV, or V, ≤1 mm ENE, contralateral LNs, close margins: reduced adjuvant RT High risk = pT1–4, N0–2 with >1 mm ENE and positive margins: adjuvant RT (standard dose)	N/A
**DELPHI**	NCT03396718	I	Accrual	Patients with resected primary and ND with indication for adjuvant therapy	Patients are randomized to: Intermediate risk = HPV + pT3 and R0 +/− 1–2 LN involvement and no ECE: 54/59.4 Gy High risk = HPV + with R1, pT4, 3+ nodes, and/or ECE: 60/66 Gy Comparative group 1 (HPV−) = 60/66 Gy Comparative group 2 (HPV+) = 60/66 Gy	N/A
	NCT03729518	II	Accrual	Resectable T1–3, N0–2c HPV-related OPSCC (7th edition)	All patients undergo TORS and ND. If post-operative pathology demonstrates <5 involved LN, patients undergo reduced adjuvant RT to nodal areas, avoiding primary site, with or without chemotherapy	N/A
	NCT02784288	I	Active, not recruiting	Potentially resectable T1–3, N0–2c HPV-related OPSCC	All patients undergo ND and biopsy of primary site. Post-operative pathology determining treatment pathway: Low risk = ≤1 LN < 6 cm, no ENE, no LVI, no PNI: TOS Intermediate risk = >/=2 LNs, presence of PNI/LVI, no ENE: RT High risk = ENE or positive margins: concurrent CRT	N/A
**Altered regimen of chemoradiotherapy**						
**NRG-HN002**	NCT02254278	II	Complete	T1–2, N1–2b or T3, N0–2b, HPV-related OPSCC (7th edition) with ≤10 PY	Patients are randomized to reduced dose 60 Gy IMRT with or without concomitant cisplatin	Group A = IMRT + C (*n* = 157) and Group B = IMRT (*n* = 149) Two-year PFS for Group A was 90.5%, and Group B was 87.6% One-year MDADI mean scores were 85.30 and 81.76, respectively. Two-year OS rates were 96.7% and 97.3%, respectively The IMRT-alone group did not meet acceptability criteria.
	NCT00606294 (pilot)NCT03323463	II	Complete	T1–2, N1–2c HPV-related OPSCC (7th edition)	Patients undergo pre-operative tumour resection and ^18^F-FMISO PET for assessment of hypoxia. No hypoxia = receive 30 Gy RT and cisplatin Hypoxia = start CRT with repeat ^18^F-FMISO PET in 1 week to reassess hypoxia If no hypoxia: 30 Gy RT with cisplatin If persistent hypoxia: 70 Gy RT with cisplatin	18 Patients included in study. 15 Patients received 30 Gy and cisplatin (6 patients had no hypoxia on initial assessment, 9 patients had no hypoxia on intra-treatment assessment) 3 Patients received 70 Gy and cisplatin Two-year locoregional control, progression-free survival, and overall survival for the de-escalated cohort per protocol were 100%, 92.9%, and 92.9%, respectively
**LCC1120**	NCT01530997	II	Complete	T0–3, N0-N2c, M0 HPV-related OPSCC with ≤10 PY (or >5 years tobacco-free if ≤30 PY) (7th edition)	All patients are treated with de-escalated IMRT (60 Gy) and reduced dose of weekly concurrent cisplatin. After completion of chemoradiotherapy, patients underwent at least ND with primary site biopsy to assess pathologic response	43/45 Patients completed the study protocol At a median 14 month from of treatment, no measurable tumor present on physical and radiologic examination in 64% of patients The pathologic complete response rate was 86% After a median 36-month follow-up, three-year locoregional control, distant metastasis-free survival, and overall survival rates were 100%, 100%, and 95%, respectively
**LCC1413**	NCT02281955	II	Complete, results not published	T0–3, N0-N2c, M0 HPV-related OPSCC with ≤10 PY (or >5 years tobacco-free if ≤30 PY) (7th edition)	All patients are treated with de-escalated IMRT (60 Gy) and reduced dose of weekly concurrent cisplatin After completion of CRT, all patients underwent PET-CT scan in place of surgery for pathologic assessment	All patients received 60 Gy IMRT (*n* = 114), 80% of the patients staged to receive chemotherapy completed at least four cycles of cisplatin and 11% received cetuximab upfront due to contraindications to cisplatin The post-treatment complete response on PET-CT was 93% at the primary site and 80% in the neck All patients with residual disease at the primary site are alive and no evidence of disease Two-year locoregional control, progression-free survival, and overall survival were 95%, 86%, and 95%, respectively
**LCCC1612**	NCT03077243	II	Active, not recruiting	T0–3, N0–2c, M0 HPV-related OPSCC (7th edition), p53 mutation status	Patients are risk stratified by their p53 mutation status and smoking history: Low risk = ≤10 PY or >10 PY without p53 mutation: 60 Gy IMRT with concurrent cisplatin High risk = >10 PY with p53 mutation: 70 Gy IMRT with concurrent cisplatin	N/A
	NCT01088802(7th edition)	II	Active, not recruiting	T1–3, any N, resectable HPV-related OPSCC	RT dose to 63 from 70 and from 58.1 Gy to 50.75 Gy	N/A
**EVADER**	NCT03822897	II	Active, not recruiting	T1–3, N0–1, M0 HPV-related OPSCC (8th edition)	Patients receive definitive RT (70 Gy) to primary site and reduced-dose elective nodal irradiation (56 Gy), with or without concurrent cisplatin	N/A
**Targeted therapy with egfr inhibitor versus cisplatin**						
**RTOG1016**	NCT01302834	III	Complete	T1–2, N2–3 or T3–4, N0–3 HPV-related OPSCC (7th edition)	Patients receive standard-dose 70 Gy IMRT and are randomized to receive concurrent cisplatin or cetuximab	Group A cetuximab (399) and Group B cisplatin (406). Median follow-up duration of 4.5 years Estimated five-year overall survival was 77.9% vs. 84.6%, respectively PFS was significantly lower in the cetuximab group as compared with the cisplatin group (hazard ratio 1.72)
**De-ESCALaTE HPV**	NCT01874171	III	Complete	T3–4, N0, T1–4, N1–3, HPV-related OPSCC with ≤10 PY (7th edition)	Patients receive standard-dose 70 Gy RT and are randomized to receive concurrent cisplatin or cetuximab	Cisplatin group (*n* = 152), cetuximab group (*n* = 152) A significant difference in two-year overall survival of 97.5% for cisplatin versus 89.4% for cetuximab, *p* = 0.001, and two-year recurrence rate of 6.0% for cisplatin versus 16.1% for cetuximab, *p* = 0.0007
**TROG12.01**	NCT01855451	III	Complete	Stage III (except T1–2, N1) or stage IV (except T3, N3 or M1) with ≤10 PY. If >10 PY, must be N0–2a (7th edition)	Patients receive standard-dose 70 Gy RT and are randomized to receive concurrent cisplatin or cetuximab	Group A cisplatin (92) and Group B cetuximab (90) There was no difference in the primary endpoint of symptom severity The T-score was 4.35 in the cisplatin arm and 3.82 in the cetuximab arm The three-year failure-free survival rates were 93% and 80%, respectively
**NRG HN005**	NCT03952585	II	Accrual	T1–2, N1 or T3, N0–2b HPV-related OPSCC with ≤10 pack year history (8th edition)	Patients are randomized to one of three arms: 70 Gy IMRT with concurrent cisplatin 60 Gy IMRT with cisplatin 60 Gy IMRT with cisplatin and nivolumab	N/A
**Neoadjuvant chemo with consolidation surgery**						
**NeCTORS**	NCT02760667	II	Accrual	Stage III-IV HPV-associated OPSCC (7th edition)	All patients undergo 3 cycles of neo-adjuvant chemotherapy with cisplatin and docetaxel and transoral surgery and selective ND	55 Patients were enrolled to undergo neoadjuvant chemotherapy and surgery, 2/55 required adjuvant CRT for unresectable positive margins following TORS, 0/55 required salvage RT for recurrence Five-year disease-free survival was 96.1% as compared with 67.6% for concurrent CRT
**Neoadjuvant chemotherapy and low dose radiotherapy**						
**E1308**	NCT01084083	II	Complete	Resectable stage III or IV HPV-related OPSCC (7th edition)	All patients undergo 3 cycles of induction chemotherapy with cisplatin, paclitaxel, and cetuximab Complete clinical response: 54 Gy adjuvant RT with weekly cetuximab Incomplete clinical response: 69.3 Gy adjuvant RT with weekly cetuximab	80 Patients were enrolled, 70% achieved a primary-site complete clinical response to induction chemotherapy, and 51 patients continued to cetuximab with IMRT 54 Gy After median follow-up of 35.4 months, two-year PFS and OS rates were 80% and 94%, respectively, for those who had complete initial response In the 69 Gy RT arm, there were higher rates of these same adverse events, with 47% suffering from mucositis and 29% having dysphagia
**Quarterback**	NCT01706939	II	Complete	Stage III-IV HPV-related OPSCC, no distant metastases, ≤20 PY (7th edition)	All patients undergo 3 cycles of induction chemotherapy with docetaxel, cisplatin, 5-fluorouracil. Patients with partial clinical response or complete clinical response were randomized (2:1) to reduced-dose IMRT (56 Gy) or standard-dose IMRT (70 Gy), with weekly carboplatin	Group A standard-dose chemoradiotherapy (8) and Group B reduced dose chemoradiation (12) Median follow up was 56 months Three-year progression-free survival was 87.5% and 83.3%, respectively Non-inferiority of reduced CRT dosages could not be demonstrated given the limited number of enrolled participants
**Quarterback II**	NCT02945631	II	Accrual	Stage III-IV, M0 HPV related OPSCC, ≤20 PY, not a current smoker (7th edition)	All patients undergo 3 cycles of induction chemotherapy with docetaxel, cisplatin, 5-fluorouracil Stratified based on response: Low risk = partial or complete clinical response: 56 Gy RT with concurrent carboplatin High risk = no response or progression: surgery or standard 70 Gy RT with concurrent carboplatin	N/A
**OPTIMA**	NCT02258659	II	Complete	T1–4, N2–3 HPV-related OPSCC (7th edition)	All patients undergo 3 cycles of induction chemotherapy with carboplatin and nab-paclitaxel Low-risk patients = ≤T3, ≤N2b, ≤10 pack-years: >50% clinical response: 50 Gy RT 30–50% clinical response: 45 Gy and concurrent paclitaxel <30% clinical response: 75 Gy and concurrent paclitaxel High risk = T4 or ≥N2c or >10 pack-years: >50% clinical response: 45 Gy and concurrent paclitaxel <50% clinical response: 75 Gy and concurrent paclitaxel	62 Patients (28 low risk/34 high risk) were enrolled Of low-risk patients, 71% received 50 Gy radiation, while 21% received 45 Gy CXRT Of high-risk patients, 71% received 45 Gy CXRT With a median follow-up of 29 months, two-year PFS and OS were 95% and 100% for low-risk patients and 94% and 97% for high-risk patients, respectively
**OPTIMA-II**	NCT03107182	II	Active, not recruiting	T3–4 or N2–3 HPV-related OPSCC (7th edition)	All patients undergo 3 cycles of induction chemotherapy with carboplatin and nab-paclitaxel, with additional nivolumab. Risk stratification based on staging and clinical response: Low-risk patients = T1–2, N2a-b >50% clinical response and TORS-eligible: TORS/neck dissection +/− reduced RT >50% clinical response and TORS-ineligible: reduced RT (50 Gy) 30–50% clinical response: 50 Gy RT with concurrent cisplatin <30% clinical response: 75 Gy and concurrent cisplatin High risk = T4, bulky N2b-2c-3, >10 pack-years: >50% clinical response: 50 Gy RT and concurrent cisplatin <50% clinical response: 75 Gy and concurrent cisplatin All patients will be offered adjuvant nivolumab for 6-months post completion of definitive therapy.	N/A
**CCRO-022**	NCT02048020/NCT01716195	II	Complete	Stage III–IV HPV-related OPSCC (7th edition)	All patients undergo 2 cycles of induction chemotherapy with paclitaxel and carboplatin. Low risk = complete clinical response or partial clinical response: 54 Gy adjuvant IMRT with concurrent paclitaxel High risk = <partial clinical response: 60 Gy adjuvant IMRT with concurrent paclitaxel	44 Patients were enrolled, 24 (55%) patients with complete or partial responses to induction chemotherapy received 54 Gy radiation, and 20 (45%) patients with less than partial responses received 60 Gy Median follow-up was 30 months. Two-year PFS was 92%

## Abbreviations

HPV, human papillomavirus; OPSCC, oropharyngeal squamous cell carcinoma; RT, radiation therapy; CRT, chemoradiotherapy; IMRT, intensity-modulated radiotherapy; TOS, transoral surgery; TORS, transoral robotic surgery; TLM, transoral laser microsurgery; ND, neck dissection; LN, lymph node; PY, pack-year; QOL, quality of life; LCR, local control rate; DMFS, distant metastases-free survival; PFS, progression-free survival; OS, overall survival; ENE, extra nodal extension; LVI, lymphovascular invasion; PNI, perineurial invasion; MDADI, MD Anderson Dysphagia Index; PET-CT, positron emission tomography-computed tomography.
